# Metformin Treatment or PRODH/POX-Knock out Similarly Induces Apoptosis by Reprograming of Amino Acid Metabolism, TCA, Urea Cycle and Pentose Phosphate Pathway in MCF-7 Breast Cancer Cells

**DOI:** 10.3390/biom11121888

**Published:** 2021-12-15

**Authors:** Thi Yen Ly Huynh, Ilona Oscilowska, Jorge Sáiz, Magdalena Nizioł, Weronika Baszanowska, Coral Barbas, Jerzy Palka

**Affiliations:** 1 Department of Medicinal Chemistry, Faculty of Pharmacy, Medical University of Bialystok, 15-089 Bialystok, Poland; ly.huynhthiyen@umb.edu.pl (T.Y.L.H.); w.baszanowska22@wp.pl (W.B.); 2 Department of Pharmaceutical and Biopharmaceutical Analysis, Faculty of Pharmacy, Medical University of Bialystok, 15-089 Bialystok, Poland; ilona.zareba@gmail.com (I.O.); magdalena.niziol@umb.edu.pl (M.N.); 3 Centre for Metabolomics and Bioanalysis (CEMBIO), Department of Chemistry and Biochemistry, Facultad de Farmacia, Universidad San Pablo-CEU, Urbanización Montepríncipe, 28660 Madrid, Spain; jorge.saizgalindo@ceu.es (J.S.); cbarbas@ceu.es (C.B.)

**Keywords:** PRODH/POX, metformin, MCF-7^crPOX^ cells, proline, glutamine, lactic acid

## Abstract

It has been considered that proline dehydrogenase/proline oxidase (PRODH/POX) is involved in antineoplastic activity of metformin (MET). The aim of this study is identification of key metabolites of glycolysis, pentose phosphate pathway (PPP), tricarboxylic acids (TCA), urea cycles (UC) and some amino acids in MET-treated MCF-7 cells and PRODH/POX-knocked out MCF-7 (MCF-7^crPOX^) cells. MCF-7^crPOX^ cells were generated by using CRISPR-Cas9. Targeted metabolomics was performed by LC-MS/MS/QqQ. Expression of pro-apoptotic proteins was evaluated by Western blot. In the absence of glutamine, MET treatment or PRODH/POX-knock out of MCF-7 cells contributed to similar inhibition of glycolysis (drastic increase in intracellular glucose and pyruvate) and increase in the utilization of phospho-enol-pyruvic acid, glucose-6-phosphate and some metabolites of TCA and UC, contributing to apoptosis. However, in the presence of glutamine, MET treatment or PRODH/POX-knock out of MCF-7 cells contributed to utilization of some studied metabolites (except glucose), facilitating pro-survival phenotype of MCF-7 cells in these conditions. It suggests that MET treatment or PRODH/POX-knock out induce similar metabolic effects (glucose starvation) and glycolysis is tightly linked to glutamine metabolism in MCF-7 breast cancer cells. The data provide insight into mechanism of anticancer activity of MET as an approach to further studies on experimental breast cancer therapy.

## 1. Introduction

Breast cancer is the most frequently diagnosed cancer in woman worldwide and has been a progressively increasing global health problem. The phenotypic characteristics can be attributed to genetic and epigenetic factors, and to nonhereditary mechanisms, such as adaptive responses or fluctuations in the tumor microenvironment signaling pathways [1]. Therefore, optimal methods of treating breast cancer must be developed to effectively cure the malignancy.

Although metformin (MET) is currently used to treat type II diabetes patients, it evokes also antineoplastic potency [2,3,4,5]. The molecular mechanism of anti-cancer activity of MET is unknown. One of the effects of MET is activation of adenosine monophosphate (AMP) kinase (AMPK) [6,7,8,9]. AMPK is activated when the AMP/ATP ratio rises. This process stimulates oxidative phosphorylation to restore normal adenosine triphosphate (ATP) levels and inhibit energy expenditure, such as cell proliferation [10,11]. the similar effects of, AMPK is regulated especially in conditions of energy shortage (e.g., glucose shortage) and hypoxia [11]. It inhibits anabolic processes and stimulates catabolism. However, MET was found to attenuate function of mitochondrial complex I resulting in decrease in ATP synthesis [12] and inhibit pyruvate kinase, impairing glucose metabolism [13]. In conditions of energy shortage and under glucose deficiency an alternative source of energy is proline, derived from protein degradation products, mainly collagen. Proline is degraded by proline dehydrogenase/proline oxidase (PRODH/POX). Of great interest is the observation that PRODH/POX is induced by AMPK [14]. Therefore, PRODH/POX could be involved in anti-cancer activity of MET.

The inhibitory role of PRODH/POX in tumor progression is well established. It has been found that PRODH/POX induces apoptosis in several cancer cell types by intrinsic or extrinsic pathway. PRODH/POX-dependent generation of ROS induces mitochondrial apoptosis (intrinsic pathway), while TRAIL (tumor necrosis factor-related apoptosis inducing ligand) and DR5 (death receptor 5) induce extrinsic pathways of apoptosis. Although, the switching mechanism for PRODH/POX-dependent intrinsic/extrinsic apoptosis is unknown, it seems that it is metabolic context dependent [15]. 

PRODH/POX (PRODH<, GenBank^TM^ NM_016335), also known as proline oxidase, is a flavin-dependent enzyme associated with the inner mitochondrial membrane [16,17]. The enzyme catalyzes conversion of proline into ∆^1^-pyrroline-5-carboxylate (P5C). During this process, electrons are transported by the electron transport chain, producing ATP, or they directly reduce oxygen, producing reactive oxygen species (ROS). In the first situation, which usually happens under low glucose stress, AMPK-dependent PRODH/POX activation produces ATP for energy supply and survival [16,18,19]. In the second one, ROS induces apoptotic pathways [20,21,22,23]. In the presence of proline, overexpression of PRODH/POX causes cytochrome c release from mitochondria to cytosol and activation of caspase-9 and caspase-3 [21,24]. Therefore, PRODH/POX may play dual role, but the mechanism that switches PRODH/POX from cancer cell growth inhibiting to growth supporting factor is unknown.

PRODH/POX cooperates with P5C reductase (P5CR) participating in proline turnover between mitochondria and cytoplasm. The conversion of proline to P5C that is shuttled between mitochondria and cytosol is coupled to glucose metabolism by pentose phosphate pathway that supports substrates for DNA biosynthesis [14,16,17,18]. It is also vital in maintenance of redox balance in a cell due to participation of NADPH/NADH in conversion of P5C to proline. Moreover, P5C is converted by P5C dehydrogenase (P5CDH) to glutamate, which is a precursor of α-ketoglutaric acid—a component of tricarboxylic acids cycle (TCA). As a result of PRODH/POX and ornithine aminotransferase (OAT) activity, proline is transformed into ornithine and enters urea cycle (UC) [16]. In view of the inhibitory role of PRODH/POX in tumor progression [19,25,26], the metabolism of proline in neoplastic cells is therefore of great importance. 

The conversion of proline into P5C by PRODH/POX is facilitated when P5C is rapidly utilized. In case it cannot be converted in mitochondria into glutamate and α-ketoglutaric acid (that enters TCA cycle), e.g., because of TCA defects, the P5C is converted to proline by P5C reductases (PYCR), mitochondrial PYCR1 or cytoplasmic PYCRL [27]. Cytoplasmic proline could be utilized for collagen biosynthesis [14] or in case of inhibition of collagen biosynthesis enters again mitochondria. Such a cycle of proline/P5C between mitochondria and cytoplasm may be responsible for ROS generation and apoptosis induction [28]. Whether MET is involved in the process requires to be explored. Another possibility is that MET-dependent activation of PRODH/POX and simultaneously inhibition of complex I of respiratory chain contributes to ROS generation instead of ATP production. 

We have suggested that MET can stimulate apoptosis in cancer cells by a cascade of processes involving the induction of AMPK, PRODH/POX and ROS generation under proline availability determined by several proline utilization/supporting processes. Proline could be derived from α-ketoglutarate, glutamic acid (Glu), glutamine (Gln) and ornithine. It links glycolysis, TCA and urea cycles. Therefore, we postulate that complex regulation of glycolysis, TCA, Urea cycles, amino acids metabolism may represent multifunctional interface that switches apoptosis or survival mode in cancer cells depending on the microenvironmental conditions. Therefore, studies on targeted metabolomic profile of MET-treated MCF-7 cells and PRODH/POX-knocked out MCF-7 cells were undertaken. It is tempted to estimate intracellular concentration of some metabolites of glycolysis, TCA, urea and pentose phosphate pathways by high performance liquid chromatography (HPLC) coupled to tandem mass spectrometry (MS) with a triple quadrupole (QqQ).

Complex analysis of the effect of MET on the metabolic profile in wild-type MCF-7 (MCF-7^WT^) breast cancer cells and the cells with knock out PRODH/POX expression (MCF-7^crPOX^) may contribute to development of knowledge on the mechanism of antineoplastic activity of MET and may help to improve experimental cancer pharmacotherapy.

## 2. Materials and Methods

### 2.1. PRODH/POX Knock out CRISPR-cas9 DNA Plasmid Purification

The sgRNAs for PRODH/POX (CRISPR All-In-One Non-Viral Vector with spCas9) were ordered by ABM Company (Richmond, Canada). The vector with expression construct was transformed into Escherichia coli DH5α and grown in Luria–Bertani (LB) media supplemented with 100 µg·mL^−1^ ampicillin at room temperature for 24 h. The targeted plasmid was extracted by a plasmid DNA purification kit (Nucleobond Xtra Midi/Maxi, MACHERY-NAREL GmbH, Düren, Germany). After being precipitated by isopropanol, the purified samples were washed by 70% ethanol solution then followed by DNA cleaning-up step by GeneMATRIX Basic DNA Purification Kit (EURX, E3545-01 protocol 1, Gdansk, Poland). The purified DNA concentration was estimated by NanoDrop™ 2000/2000c Spectrophotometers (Thermo Fisher Scientific, Waltham, MA, USA).

### 2.2. Transfection into MCF-7 Breast Cancer Cell Line

MCF-7 breast cancer cells were cultured in the complete growth medium, DMEM 1X (Gibco) containing 4.5 gL^−1^ glucose, L-glutamine and pyruvate supplemented with 10% Fetal Bovine Serum (FBS) qualified (Gibco), 1% penicillin/streptomycin (Invivogen) at 37 °C in 5% CO_2_. The cells were then seeded into 6-well plates to reach 70–90% confluency. The amount of plasmid in the experiment was tested from 1 to 2 µg per well. Lipofectamine 2000 (Invitrogen, Thermo Fisher Scientific, Waltham, MA, USA) was used as a transfection reagent.

Prior to transfection, the plasmid was diluted with 50 µL of medium A, DMEM 1X (Gibco) containing 4.5 gL^−1^ glucose, L-glutamine and pyruvate supplemented with 1% penicillin/streptomycin (Invivogen). 

The transfection solution containing 805.4 µL of medium A and 194.6 µL of lipofectamine reagent were gently mixed then incubated at room temperature for 5 min before aliquoting 60 µL of the solution into a vial containing the diluted plasmid solution. The mixture of diluted plasmid and transfection solution was mixed gently then incubated at room temperature for 20 min.

The testing cells were washed by PBS 1X (sterile phosphate buffered saline 1X, Gibco) and freshly added with 1 mL of medium A. After 20-min incubation, the mixture of plasmid and transfection reagent were slowly added to cells then incubated at 37 °C in 5% CO_2_ overnight. The following day, the transfected cells were selected in the complete growth medium with 1 µg·mL^−1^ of puromycin (Sigma-Aldrich, St. Louis, MI, USA) in the same culture conditions for 10 days. The expression of PRODH/POX in transfected cells was checked by Western blot. Based on the results of expression level between wild-type MCF-7 cells and transfected MCF-7 cells, the PRODH/POX knock out MCF-7 cell line was selected for further stable clone generation. The process of the stable clone generation was manipulated with a serial dilution of the selected cells in the culture media how to obtain 0.7 cell per well in a 96-well plate. The screening steps were done with a random selection of cell clones. The PRODH/POX silencing in cell clones were checked by Western blot using an anti-PRODH/POX antibody (Santa Cruz, Dallas, TX, USA). The level of PRODH/POX knock down is presented in Supplementary Materials (Figure S1). The PRODH/POX knock out MCF-7 cells defined as MCF-7^crPOX^ cells were banked for further experiments.

### 2.3. Cell Culture

Wild type MCF-7 (MCF-7^WT^) cells and PRODH/POX-knocked out cells (MCF-7^crPOX^) cells were cultured in DMEM 1X (Gibco) containing 4.5 gL^−1^ glucose, L-glutamine and pyruvate supplemented with 10% Fetal Bovine serum (FBS) qualified (Gibco), 1% penicillin/streptomycin (Invivogen) at 37 °C in 5% CO_2_. The assay media used in this approach were DMEM 1X containing 4.5 gL^−1^ glucose, L-glutamine and pyruvate supplemented with 1% Penicillin/streptomycin (Gibco); DMEM 1X (Gibco) containing 4.5 gL^−1^ glucose, 0.11 gL^−1^ sodium pyruvate, without L-glutamine supplemented with 1% penicillin/streptomycin (Gibco); DMEM 1X (Gibco) containing 1.0 gL^−1^ glucose, pyruvate, without L-glutamine supplemented with 1% Penicillin/streptomycin (Gibco). 

The cells were seeded into Petri dishes to obtain approximately 10 million cells per plate. After that the cells were treated with/without Metformin in 3 different assay media overnight. Fifty testing samples of Metformin untreated/treated wild type MCF-7 cells and MCF-7^crPOX^ cells in different cultured conditions were assigned into 10 different groups were listed in Table S1 (Supplementary Materials). Every group contains 5 replicates of testing samples.

### 2.4. Metabolite Extraction

After treatment overnight, a sample (approximately 10–20 million cells) was collected in a vial without trypsinizing. The testing cells were washed by PBS 1X (Gibco) before scraping to collect into vial then stored at −80 °C. For extraction, 250 µL of acetonitrile (ACN) (Merck, Darmstadt, Germany) was added into a vial. The cell suspension was sonicated at 60 kHz, for 5 s per time, then place a vial on ice for minute. This step was repeated 4 times. The cell debris was separated by centrifugation (Eppendorf Centrifuge 5415R, Hamburg, Germany) at 16,000× *g* at 4 °C for 15 min. Supernatants (50 µL) was injected to LC-QqQ for targeted approaches. 

### 2.5. Targeted Metabolomics Quantitative Analysis (LC-MS/MS(QqQ))

This study focused on several metabolites involved in Glycolysis, TCA cycles, pentose phosphate pathway, urea cycles and several key amino acids in PRODH/POX-dependent pathways. Testing metabolites are summarized in Table S2 (Supplementary Materials). All stock solution of reference metabolites were prepared in acetonitrile to obtain 1000 ppm (mg·mL^−1^). LC-MS/MS analysis was performed using an Agilent 1200 LC coupled to an Agilent 6470 Triple quadrupole (Agilent Technologies, Santa Clara, CA, US) with an InfinityLab Poroshell 120 HILIC-Z column (Agilent Technologies, Santa Clara, CA, US) for (hydrophilic liquid chromatography (HILIC) interaction. The platform was operated in a multiple reaction monitoring (MRM) in negative mode using an electrospray ionization (ESI) source. The optimized transition of amino acid metabolites is listed in Table S3 (Supplementary Materials). The injection volume was 2 µL. Mobile phase A was 10 mM ammonium acetate adjusted to pH = 9 with ammonia, with 2.5 mM InfinityLab deactivator additive (Agilent, P-N. 5191-4506). Mobile phase B was 10 mM ammonium acetate adjusted to pH = 9 in H_2_O/ACN (15:85, *v*/*v*) with 2.5 mM of the same deactivator. The flow was constant at 0.250 mL/min. The chromatographic gradient is described in reference [29]. 

### 2.6. Data Pre-Treatment

After data acquisition, all chromatograms were inspected in MassHunter Qualitative analysis navigator 8.0 (Figure S7, Supplementary Materials). Accurate peak integration was performed by using Mass Hunter Quantitative analysis (for QqQ) version 8.0 (Figure S8, Supplementary Materials). Stock solutions at different concentrations, ranging from 1 ppb to 20,000 ppb were prepared and were used to construct calibration curves that covered the range of each metabolite. The quantitation was performed in Excel.

### 2.7. Cell Lysate Preparation

Cells were cultured in FBS-free DMEM with or without glutamine and MET (20 mM) for 24 h. The procedure for harvesting the cells was performed as previously described [30]. The supernatant was aliquoted and stored at −80 °C. Protein concentration was measured using the Pierce BCA assay kit (Thermo Fisher Scientific, Waltham, MA, USA).

### 2.8. Cell Proliferation Assay

The proliferation of MCF-7 and MCF-7^crPOX^ cells was evaluated using CyQUANT^®^ Cell Proliferation Assay (Thermo Fisher Scientific, Waltham, MA, USA) according to the manufacturer’s procedure. The cells were cultured in glutamine free or glutamine containing DMEM and treated with MET (20 mM) for 24 h. The read was performed on TECAN Infinite^®^ M200 PRO (Tecan Group Ltd., Männedorf, Switzerland) at 480 and 520 nm as excitation and emission wavelengths, respectively. The results were presented as a percent of the control value.

### 2.9. Cell Cycle Analysis

The cells were trypsinized and centrifuged (5 min, 500× *g*) followed by washing twice with phosphate-buffered saline (PBS). The suspended pellet (500 µL PBS) was fixed in 70% ethanol (4.5 mL) and stored (4 °C) until the day of analysis. After centrifugation (5 min, 500× *g*), ethanol-fixed cells were mixed with Solution 3 (ChemoMetec, Allerod, Denmark), incubated (37 °C, 5 min), and analyzed with an image cytometer (NC-3000, ChemoMetec, Allerod, Denmark).

### 2.10. Western Immunoblotting

Western blot analysis was carried out as described by Misiura et al. [30]. The membranes were incubated with primary antibodies diluted 1000 times in 5% bovine serum albumin (Sigma Aldrich, Saint Louis, MO, USA) in TBS-T (20 mM Tris, 150 mM NaCl, 0.1% Tween-20, pH 7.6). Anti-PARP, anti-AMPK and anti-caspase-7 and anti-GAPDH, were purchased from Cell Signaling Technology, Danvers, MA, USA; anti-PRODH/POX from St John’s Laboratory, London, UK), followed by incubation with alkaline phosphatase-linked goat anti-rabbit or anti-mouse antibodies (dilution: 1:10,000 in 5% non-fat dried milk (Santa Cruz Biotechnology, Dallas, TX, USA) in TBS-T; Sigma Aldrich, Saint Louis, MO, USA). The bands’ intensities were semi-quantitatively measured in ImageJ software (https://imagej.nih.gov/ij/, accessed on 27 October 2021). All experiments were run at least in triplicates.

### 2.11. Statistical Analyses

#### 2.11.1. Targeted Analysis

GRAGHPAD PRISM version 9.0 was used to perform Mann–Whitney tests using the five replicates per group included in this study. Supervised Orthogonal Partial Least Square-Discriminant analysis (OPLS-DA) in SIMCA was applied for multivariate statistics. The volcano plots were built in order to obtain variable importance in the projection (VIP) values and corrected *p*-values (p(corr)). Those metabolites with VIP > 1.00, q ≤ 0.050 and absolute p(corr) ≥ 0.30 were considered as significant. The percentages of change reflecting the difference of each metabolite level between groups were also calculated.

#### 2.11.2. Biological Analysis

All experiments were carried out in duplicates and the experiments were repeated at least three times. Data are shown as a mean ± standard error (SEM). For statistical calculations, one-way analysis of variance (ANOVA) with Dunnett’s correction and *t*-test were used. Statistical analysis was performed using GraphPad Prism 5.01 (GraphPad Software, San Diego, CA, USA). Statistically significant differences were marked as * *p* < 0.05, ** *p* < 0.01, *** *p* < 0.001 and **** *p* < 0.0001.

## 3. Results

### 3.1. Metformin Inhibits MCF-7 Cell Proliferation and Induces Apoptosis 

**Figure 1:**
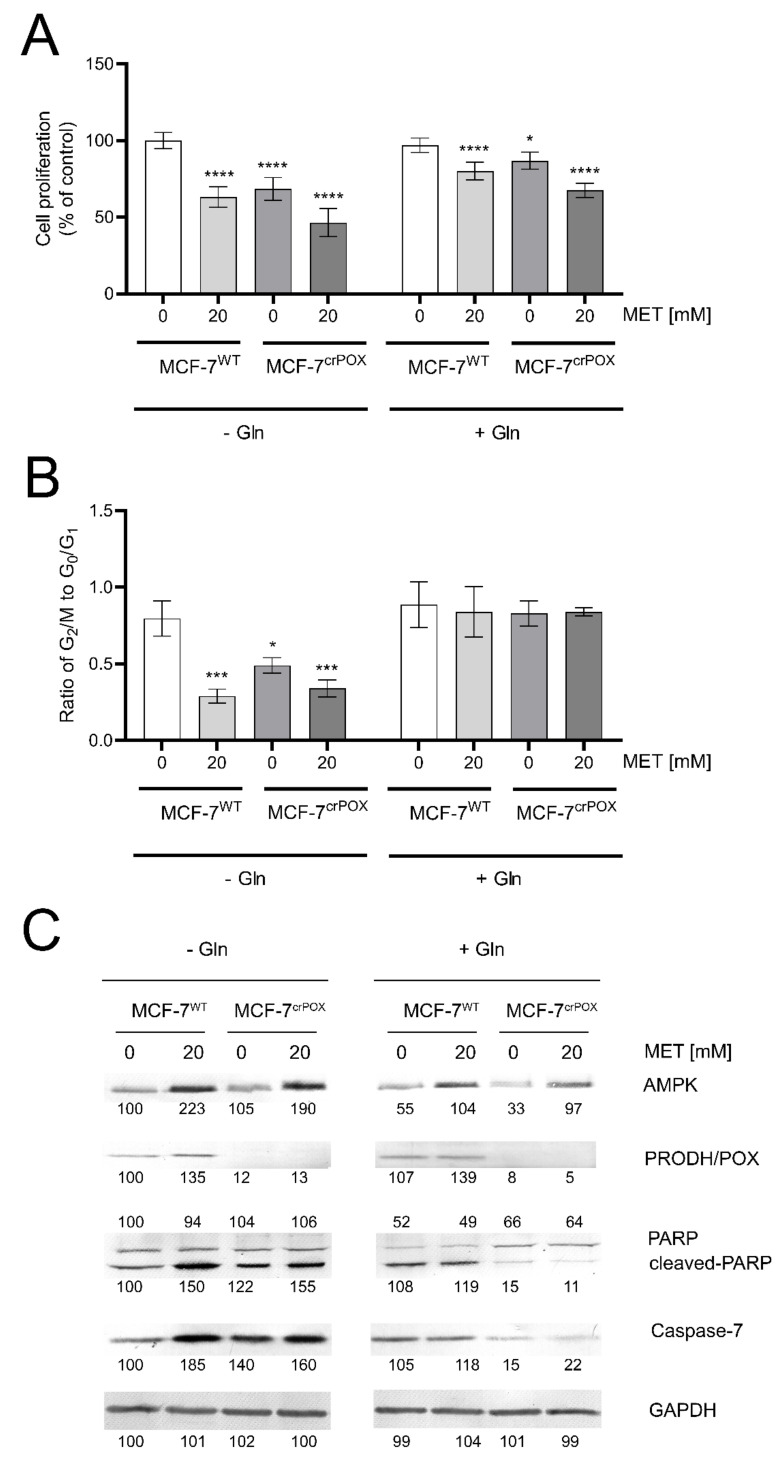
Cell proliferation (**A**) the ratio of cell percentage in G_2_/M to G_0_/G_1_ phase. (**B**) Western blot for AMPK, PRODH/POX, PARP and caspase 7. (**C**) in metformin (MET, 20mM) treated MCF-7^WT^ and PRODH/POX-knock out MCF-7^crPOX^ cells cultured in medium with or without glutamine (Gln) for 24 h. The mean values ± standard error (SEM) from 3 experiments done in duplicates are presented at * *p* < 0.05, *** *p* < 0.001, and **** *p* < 0.0001. Representative Western blot images were shown. Supplementary Materials contain statistical analysis of the evaluated proteins (Figures S3–S6). The percentage of cells in G0/G1, S and G2/M phases of the cell cycle of MCF-7^WT^ and MCF-7^crPOX^ cells treated with metformin with or without Gln (Figure S2).

MCF-7 breast cancer cell line (MCF-7^WT^) and the corresponding MCF-7 cell line with PRODH/POX-knock out (MCF-7^crPOX^) were treated with metformin (MET, 20 mM) for 24 h in medium with or without glutamine. MET-treatment of MCF-7^WT^ cells or PRODH/POX-knock out of the cells contributed to decrease in cell proliferation, when incubated in medium with or without glutamine (Figure 1A). MET potentiated inhibition of cell proliferation in both cell lines. However, this process was more pronounced in the absence of glutamine. The data were corroborated by the ratio of dividing cells to non-dividing cells (the percentage of cells in G2/M phase to G0/G1 phase). As presented on Figure 1B, MET-treatment and PRODH/POX knock out strongly inhibited proliferation of MCF-7 cell cultured in glutamine free medium, while in the presence of glutamine there was no effect on the process. 

MET induced expression of AMPK in both cell lines (Figure 1C). In the cells cultured in the absence of glutamine this process was more pronounced. The expression of PRODH/POX was also increased in MET-treated MCF-7^WT^ cells cultured in medium with or without glutamine, while in MCF-7^crPOX^ cells, the PRODH/POX was not detected and MET did not affect its expression. MET increased the expression of cleaved PARP and Caspase-7 in both cell lines when cultured in glutamine free (-Gln) medium. Interestingly, PRODH/POX knock out by itself also increased expressions of cleaved PARP and Caspase-7 in MCF-7^crPOX^ cells, compared to MCF-7^WT^ cells, when cultured in glutamine free medium. However, in the presence of glutamine (+Gln), MET did not affect very low expression of the proteins in both studied cell lines (Figure 1 C). 

### 3.2. Targeted Metabolic Profiles of Some Metabolites of Glycolysis, Pentose Phosphate Pathway, TCA and Urea Cycles in PRODH/POX-Knock out of MCF-7 Cells (MCF-7^crPOX^) and Wild Type MCF-7 Cells (MCF-7^WT^) Cultured in Glutamine (Gln) Free Medium

PRODH/POX-knock out of MCF-7 cells (MCF-7^crPOX^) contributed to drastic increase in intracellular glucose (GLC) and pyruvic acid (PYR) concentrations (about 12- and 17-fold, respectively) and about 2-fold increase in lactic acid (LA) concentration, as compared to MCF-7 wild type cells (MCF-7^WT^). It was accompanied by total decrease in the concentrations of phospho-enol-pyruvic acid (PEP) and glucose 6-phosphate (G-6-P), 6-Phospho-gluconic acid and significant decrease in the concentrations of all TCA cycle and urea cycle metabolites as well as glutamine (Gln) and glutamic acid (Glu), without effect on proline (Pro) concentration in PRODH/POX-knocked out MCF-7 cells (Table 1).

The data suggest that PRODH/POX-knock out contributes to inhibition of GLC, LA and PYR consumption while PEP and G-6-P as well as some TCA and urea cycles metabolites are utilized in these conditions. Pro is not significantly affected.

### 3.3. Targeted Metabolic Profiles of Some Metabolites of Glycolysis, Pentose Phosphate Pathway, TCA and Urea Cycles in Metformin (MET) Treated Wild Type MCF-7 Cells (MCF-7^WT+MET^) and in MCF-7^WT^ Cells Cultured in Gln Free Medium

Metformin-treatment of MCF-7^WT^ (MCF-7^WT+MET^) contributed to drastic increase in GLC, PYR, LA compared to control MCF-7^WT^ cells. It was accompanied by decrease in PEP (insignificantly), G-6-P and some TCA metabolites concentrations, compared to MCF-7^WT^. Of interest is no effect on Pro concentration in MCF-7^WT+MET^ cells, compared to MCF-7^WT^ cells (Table 2).

The data suggest that MET contributes to decrease in GLC, PYR and LA consumption while PEP and G-6-P as well as some TCA metabolites are utilized in these conditions, as compared to MCF-7^WT^ cells. Pro is not significantly affected.

### 3.4. Targeted Metabolic Profiles of Some Metabolites of Glycolysis, Pentose Phosphate Pathway, TCA and Urea Cycles in MCF-7^crPOX^ Treated with MET (MCF-7^crPOX+MET^) and in MCF-7^WT^ Cultured in Gln Free Medium 

MET treatment of MCF-7^crPOX^ cells (MCF-7^crPOX+MET^) contributed to increase in intracellular GLC, PYR (about 26- and 44-fold, respectively) and drastic increase in LA (about 4-fold) concentrations, as compared to MCF-7 wild type cells (MCF-7^WT^). It was accompanied by total decrease in the concentrations of PEP and G-6-P and significant decrease in the concentrations of several TCA cycle and ornithine in MCF-7^crPOX+MET^ compared to MCF-7^WT^cells (Table 3).

The data suggest that MET treatment of PRODH/POX-knock out MCF-7 cells (MCF-7^crPOX+MET^) contributes to inhibition of GLC, LA and PYR consumption while PEP and G-6-P and some TCA and urea cycles metabolites are utilized in these conditions.

### 3.5. Targeted Metabolic Profiles of Some Metabolites of Glycolysis, Pentose Phosphate Pathway, TCA and Urea Cycles in PRODH/POX-Knock out of MCF-7 Cells (MCF-7^crPOX^) and Wild Type MCF-7 Cells (MCF-7^WT^) Cultured in Medium Containing Gln

The result showed that although differential levels of metabolites between groups were not statistically different, PRODH/POX- knock out of MCF-7 cells (MCF-7^crPOX^) contributed to increase in intracellular GLC (insignificantly), slight increase in PYR concentrations and decrease in concentrations of PEP, G-6-P, some TCA cycle and urea cycle metabolites as well as Gln and Glu, without effect on Pro concentration in PRODH/POX- knocked out MCF-7 cells as compared to MCF-7^WT^ (Table 4). 

The data suggest that the PRODH/POX-knocked out cells cultured in the presence of Gln utilize all studied metabolites, while saves consumption of GLC in these conditions. Pro is not significantly affected.

### 3.6. Targeted Metabolic Profiles of Some Metabolites of Glycolysis, Pentose Phosphate Pathway, TCA and Urea Cycles in MET Treated Wild Type MCF-7 Cells (MCF-7^WT+MET^) and in MCF-7^WT^ Cells Cultured in Medium Containing Gln

The results of high percentage change indicated that Metformin-treatment of MCF-7^WT^ (MCF-7^WT+MET^) contributed to drastic increase in GLC (about 11-fold), PYR, Gln, Glu and decrease in LA, G-6-P, Orn and some metabolites of TCA cycle. However, concentrations of Pro and Arg were not much affected, compared to MCF-7^WT^ (Table 5).

The data suggest that MET treated cells (MCF-7^WT^) cultured in the presence of Gln contributes to inhibition of utilization of GLC, PYR, Gln and Glu, while utilizes TCA metabolites and lactic acid and only slightly affect Pro and some urea cycle metabolites, as compared to MCF-7^WT^ cells.

### 3.7. Targeted Metabolic Profiles of Some Metabolites of Glycolysis, Pentose Phosphate Pathway, TCA and Urea Cycles in MCF-7^crPOX^ Treated with MET (MCF-7^crPOX+MET^) and in MCF-7^WT^ Cultured in the Medium Containing Gln

MET treatment of MCF-7^crPOX^cells (MCF-7^crPOX+MET^) in the presence of Gln contributed to drastic increase in concentration of GLC (about 18-fold), not significant increase in PYR and Pro and total decrease in PEP, G-6-P and significant decrease in concentration of TCA, urea cycle metabolites and a slight decrease in LA. Interestingly, Glu concentration was significantly decreased, compared to MCF-7^WT^ (Table 6).

The data suggest that MET treatment of MCF-7^crPOX^ cells (MCF-7^crPOX+MET^) cultured in the presence of Gln contributes to inhibition of GLC utilization while induce utilization of TCA and urea cycle metabolites and LA, without significant effect on Pro concentration, as compared to MCF-7^WT^ cells.

## 4. Discussion

Epidemiological evidence suggests that therapy with the metformin is associated with decreased risk of certain cancers, such as colon, liver, lung as well as decreased cancer mortality [31]. However, there is some discrepancy between these studies. Some data show beneficial effect of metformin in cancer treatment with reduced mortality [32,33,34,35], while others fail to document such beneficial effects [31,36]. It suggests the presence of a specific molecular signature of cancer that increases its susceptibility to the antineoplastic effects of metformin. Therefore, we try to recognize the molecular signature by metabolomic approach.

Metabolomic analyses are promising approaches for identification of specific abnormalities in cancer metabolic pathways that could be considered as a potential target for cancer therapy. Similarly, metabolomic analyses of cancer cells that are treated with compounds of potential antineoplastic activity could identify mechanism of their action. In present study, analysis of some metabolites (targeted metabolomics) of glycolysis, TCA, Urea cycle, PPP and proline convertible amino acids (glutamine, glutamate, ornithine, α-ketoglutarate) was performed in breast cancer cells that have been treated with MET. It has been considered that MET induces reprogramming of energetic metabolism in such a way that instead of glucose facilitate degradation of proline by PRODH/POX, as an alternative source of energy. Therefore, studies on PRODH/POX-knocked out MCF-7 cells were also performed.

**Figure 2:**
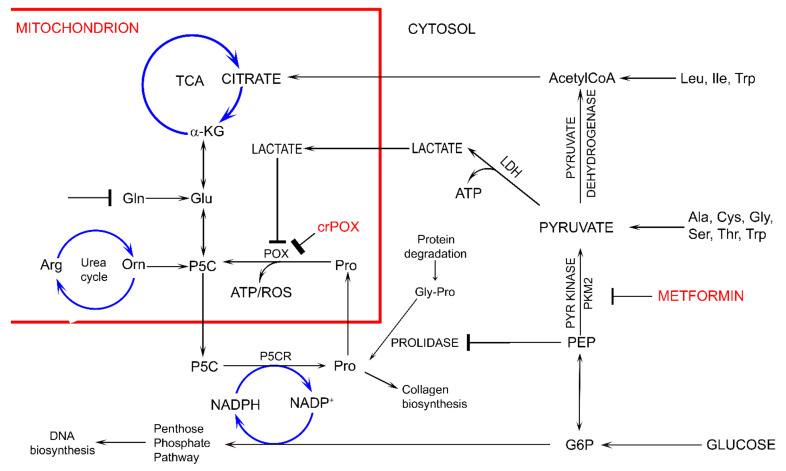
The potential effect of metformin (MET) on complex regulatory mechanisms of PRODH/POX-dependent apoptosis/survival linking glycolysis, TCA, urea cycles, pentose phosphate pathway, proline cycle (synthesis and degradation), collagen biosynthesis and degradation and prolidase. α-KG—α-ketoglutarate, AcetyloCoA—acetyl coenzyme A, Ala—alanine, Arg—arginine, ATP—adenosine triphosphate, crPOX—CRISPER for POX, Cys—cysteine, Gln-glutamine, Glu- glutamic acid, G6P—glucose-6-phosphate, Gly—glycine, Gly-Pro—glycyl-proline, Ile—isoleucine, LDH—lactate dehydrogenase, Leu—leucine, NADP^+^—nicotinamide adenine dinucleotide phosphate, NADPH—reduced form of NADP^+^, PEP—phosphoenolpyruvate, PYR kinase—pyruvate kinase, Orn—ornithine, PKM2—pyruvate kinase M2, Pro—proline, POX—proline dehydrogenase/oxidase, P5CR—1-pyrroline-5-carboxylate reductase, P5C—1-pyrroline-5-carboxylate, ROS—reactive oxygen species, Ser—serine, TCA—tricarboxylic acid cycle, Thr—threonine, Trp—tryptophan.

Interestingly, in conditions of Gln absence, MET treatment of MCF-7 cells as well as MCF-7 PRODH/POX-knocked out cells contributed to similar inhibition of glycolysis (increased intracellular concentration of GLC, PYR and LA) and utilization (decreased concentration) of PEP, G-6-P and some metabolites of TCA and urea cycle, without significant effect on Pro level, as compared to control MCF-7^WT^ cells. The functional significance of the phenomenon is activation of apoptosis. However, in the presence of Gln, MET treatment of MCF-7 cells as well as MCF-7 PRODH/POX-knocked out cells contributed to utilization of some studied metabolites, (except GLC) and creation of pro survival phenotype of MCF-7 cells cultured in these conditions. It suggests that glycolysis is linked to glutamine and proline metabolism. In fact, glycolysis is quiescent not only in MET treated MCF-7 cells but also in non-treated PRODH/POX-knocked out MCF-7 cells. It seems that in both cases there is metabolic glucose starvation and the cells favor Gln as the source of alternative metabolic energy over glucose. The glucose-independence in these conditions suggests uncoupled glycolysis and TCA cycle that might be the sign of MET-dependent rewiring of energetic metabolism. The possible mechanism of this process could involve MET-dependent inhibition of pyruvate kinase attenuating glucose utilization and subsequently TCA metabolism and P5C synthesis with further consequences on proline cycle and PPP (Figure 2). The link could be also at the level of lactate dehydrogenase (LDH) converting PYR to LA and coupled to redox state by regeneration of NAD for stimulation of glycolysis and simultaneously preventing GLC processing in TCA cycle.

Cancer cells are characterized by enhanced consumption of glucose-yielding lactate during aerobic glycolysis. The phenomenon known as a Warburg effect ensures rapid production of ATP from glucose to support cancer cell proliferation [37,38]. Though the process of ATP production from glucose by Warburg effect is less efficient than during mitochondrial oxidative phosphorylation, the conversion of pyruvate into lactate ensures high NAD+/NADH ratio that accelerate glycolysis. For a long time, Warburg effect has been considered as an effect of impairment of oxidative phosphorylation, but in recent decades it has been documented that the mechanism underlying cancer metabolic reprogramming is much more complex [39]. It is well established that Warburg effect contributes to depletion of TCA cycle and augmentation of glutaminolysis, feeding in this way TCA by glutamine metabolites, as, e.g., α-ketoglutarate [40]. This process is significantly impacted by non-essential amino acids as proline, ornithine and glutamate. They are interconvertible with intermediate of P5C, linking TCA and urea cycles with glutamine metabolism. Particularly, proline could serve as an alternative source of energy. Large quantity of proline comes from protein degradation, mostly from the most abundant extracellular protein, collagen. Deregulation of energetic metabolism in cancer cells due to Warburg’s effect facilitates protein degradation as an alternative source of energy.

**Figure 3:**
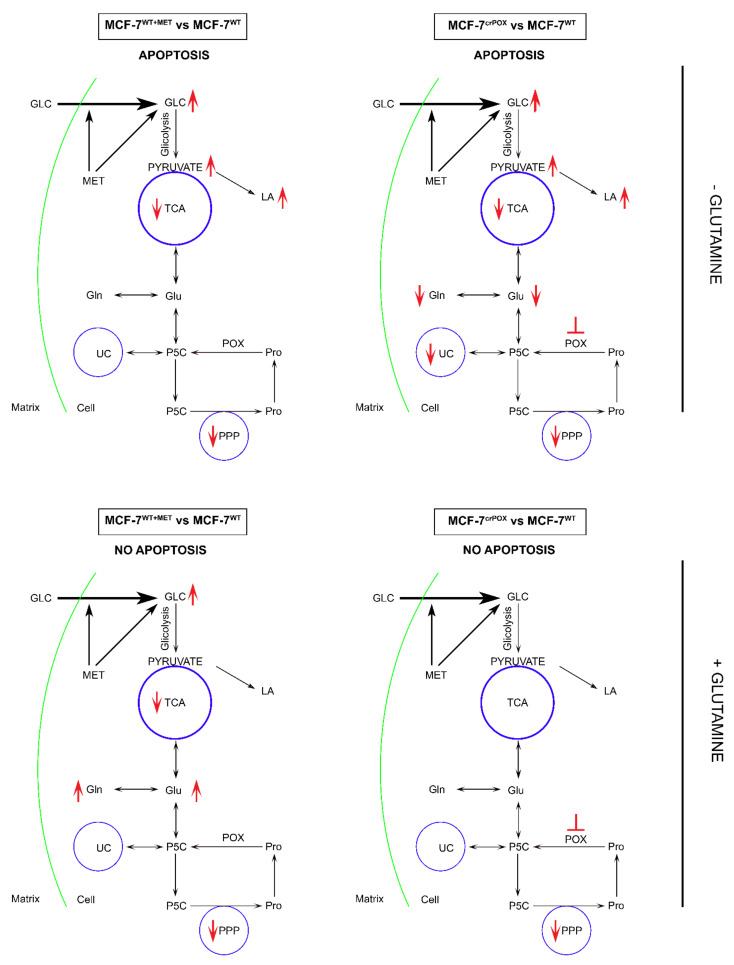
The functional significance of metformin (MET) and PRODH/POX knock-down on complex regulatory mechanisms driving PRODH/POX-dependent apoptosis/survival in wild-type MCF-7 cells (MCF-7^WT^) and PRODH/POX-knock out MCF-7 cells (MCF-7^crPOX^), cultured in the presence or absence of Gln. crPOX—CRISPER for POX, GLC—glucose, Gln-glutamine, Glu- glutamic acid, LA—lactate dehydrogenase, Pro—proline, POX—proline dehydrogenase/oxidase, PPP—pentose phosphate pathway, P5C—1-pyrroline-5-carboxylate, TCA—tricarboxylic acid cycle, UC—urea cycle.

Several studies showed that proline concentration is increased in cancer cells [41,42]. Both hypoxia [43] and glucose depletion [14] were found to induce activity of metalloproteinases, MMP-2 and -9, suggesting the mechanism for the increase in cellular proline concentration. When glucose supply is limited, cancer cells may select proline as an alternative energy source, since proline has an advantage over fatty acids and glutamine, which like glucose require delivery by the circulation. Therefore, proline may represent energy sense molecule and energy substrate. Especially, under glucose deprivation, in order to maintain the cell survival, proline interconvertible amino acids: glutamate, α-ketoglutarate and ornithine may serve as alternative sources of energy. They are substrates for production of P5C that links TCA, urea cycles and glutamine metabolism. P5C as a product of proline conversion by PRODH/POX is of special interest. P5C and proline circulate between mitochondria and cytoplasm. Conversion of P5C into proline is catalyzed by P5C reductase (P5CR). The shuttle is known as a “proline cycle”. It is coupled to pentose phosphate pathway (PPP) producing nucleotides for DNA biosynthesis. The data presented in this paper suggest tight correlation between glycolysis, proline metabolism by PRODH/POX and PPP. PRODH/POX-knock out of MCF-7 cells or treatment of the cells with MET inhibited glycolysis (increase in intracellular GLC concentration), and attenuated PPP and TCA pathways (decrease in the concentration of metabolites) when cultured in Gln free medium. In the presence of Gln, the cells similarly inhibited GLC utilization however, differentially affected LA utilization. PRODH/POX-knocked out MCF-7 cells utilized LA, while treated with MET inhibited LA utilization in these conditions. It suggests that inhibition of glycolysis in PRODH/POX-knocked out MCF-7 cells and MET treated cells is affected by Gln. Moreover, PRODH/POX-knock out MCF-7 cells that has been treated with MET in Gln free medium inhibited utilization of GLC and LA, while in the presence of Gln induced utilization of LA. It suggests synergistic effects of PRODH/POX-knock out and MET treatment on inhibition of glycolysis and the role of Gln in stimulation of LA utilization in these cells. Therefore, the similar effects of metformin treatment and knockout of PRODH/POX on breast cancer cellular metabolism could be explained at the level of multidirectional regulatory mechanisms including glycolysis, TCA cycle, urea cycle, proline cycle and amino acid metabolism, as shown in Figure 2. It seems that the key metabolite is P5C. Since metformin inhibits pyruvate kinase, it inhibits glucose utilization and subsequently down-regulate TCA cycle and P5C synthesis with further consequences on proline cycle and pentose phosphate pathway. The similar effect could be achieved when PRODH/POX is knocked out. The functional significance of the processes (activation of apoptosis) was found in MCF-7 cells cultured in glutamine free medium. However, when the cells were cultured in the presence of glutamine (provider of P5C) apoptosis did not occur. The potential mechanism of this processes is outlined in Figure 3.

Recently we have found that silencing of PRODH/POX induced autophagy while overexpression of prolidase and inhibition of collagen biosynthesis contributed to increase in intracellular proline concentration and PRODH/POX-dependent autophagic cell death in MCF-7 cells [44]. It has been suggested that up-regulation of PRODH/POX by PPAR-gamma ligands could induce apoptosis in cancer cells [45]. Since LA generated in cancer cells due to Warburg effect inhibits PRODH/POX [46], limiting its function (apoptosis/autophagy), it seems that inhibition of Warburg effect (lactate production, e.g., by metformin) contributed to up-regulation of PRODH/POX -induced apoptosis in cancer cells. In fact, inhibiting LA generation in cancer cells by MET attenuated cancer cell growth and survival [47,48,49]. The data are also supported by studies showing that PRODH/POX is induced by AMP-activated protein kinase (AMPK)-dependent pathways [16] and phosphorylated-AMPK was upregulated following glycolysis inhibition by 3-bromopyruvate (3-BP) treatment [50].

We suggest that MET inhibits glycolysis and TCA cycle leading to glucose starvation, ATP depletion, facilitating apoptosis. Similar mechanism was presented for 3-bromopyruvate, inhibitor of pyruvate dehydrogenase [51]. Of great importance is its potential to affect pentose phosphate pathway (PPP) that produce reducing potential and nucleotides for DNA synthesis [52]. Since PPP is directly coupled to glycolysis, any changes in glycolytic pathway may affect NADPH production and DNA biosynthesis. The hypothesis is outlined in Figure 2.

## 5. Conclusions

Metformin treatment of MCF-7 breast cancer cells or PRODH/POX-knock out of the cells induces apoptosis by reprograming of amino acid metabolism, TCA, Urea cycle and pentose phosphate pathway in the cells. Metabolomic analyses in the cells cultured with or without Gln suggest that glycolysis is tightly linked to Gln and Pro metabolism. In the absence of Gln, MET-treatment or PRODH/POX-knock out contributed to GLC starvation and apoptosis in MCF-7 cells as outlined in Figure 3. This knowledge provide insight into mechanism of anticancer activity of MET as an approach to further studies on experimental breast cancer therapy.

## Figures and Tables

**Table 1:** Targeted metabolic profiles of some metabolites of glycolysis, pentose phosphate pathway, TCA and urea cycles in PRODH/POX-knock out of MCF-7 cells (MCF-7^crPOX^) and wild type MCF-7 cells (MCF-7^WT^) cultured in glutamine (Gln) free medium. AA—amino acids, PPP—pentose phosphate pathway, TCA—tricarboxylic acids cycle. ↑ —significant increase in the concentration of studied compound in MCF-7^crPOX^ cells vs. MCF-7^WT^, ↓ —significant decrease in the concentration of studied compound in MCF-7^crPOX^ cells vs. MCF-7^WT^.

Relevant Metabolic Pathways	Metabolites	Conc. Average (ppb)		% Change (MCF-7^crPOX^ vs. MCF-7^WT^)	*P* Value (Mann–Whitney)	MCF-7^crPOX^
		MCF-7^WT^	MCF-7^crPOX^			
Glycolysis	Pyruvic acid	6.5	117.2	1712.0	0.010	↑
	Glucose	11.2	150.8	1252.0	0.010	↑
	Phospho-enolpyruvic acid	6449.0	0.0	−100.0	0.010	↓
PPP	Glucose 6-phosphate	328.6	0.0	−100.0	0.010	↓
	6-Phospho-gluconic acid	269.9	10.9	−96.0	0.010	↓
TCA	Malic acid	1126.1	77.4	−93.1	0.010	↓
	Succinic acid	250.5	131.5	−47.5	0.159	
	Fumaric acid	336.2	27.5	−91.8	0.010	↓
	cis-Aconitic acid	43.7	4.2	−90.3	0.010	↓
	Citric acid	6425.6	0.0	−100.0	0.010	↓
	alpha-Ketoglutaric acid	222.3	97.1	−56.3	0.019	↓
Urea Cycle	Citrulline	10.3	4.6	−55.1	0.035	↓
	Arginine	14,526.1	437.6	−97.0	0.010	↓
	Ornithine	2626.1	0.0	−100.0	0.010	↓
AA	Proline	2642.0	2694.3	2.0	0.841	
	Glutamine	31.0	20.3	−34.5	0.010	↓
	Glutamic acid	410.2	155.9	−62.0	0.010	↓
Additional	Lactic acid	4613.3	13,347.5	189.3	0.010	↑
	Fructose	2.3	34.6	1384.5	0.010	↑

**Table 2:** Targeted metabolic profiles of some metabolites of glycolysis, pentose phosphate pathway, TCA and urea cycles in metformin (MET) treated wild type MCF-7 cells (MCF-7^WT+MET^) and in MCF-7^WT^ cells cultured in Gln free medium. AA—amino acids, PPP—pentose phosphate pathway, TCA—tricarboxylic acids cycle. ↑ —significant increase in the concentration of studied compound in MCF-7^WT+MET^ cells vs. MCF-7^WT^, ↓ —significant decrease in the concentration of studied compound in MCF-7^WT+MET^ cells vs. MCF-7^WT^.

Relevant Metabolic Pathways	Metabolites	Conc. Average (ppb)		% Change (MCF-7^WT+MET^ vs. MCF-7^WT^)	*P* Value (Mann–Whitney)	MCF-7^WT+MET^
		MCF-7^WT^	MCF-7^WT+MET^			
Glycolysis	Pyruvic acid	6.5	227.9	3423.5	0.038	↑
	Glucose	11.2	115.8	938.0	0.038	↑
	Phospho-enolpyruvic acid	6449.0	417.1	−93.5	0.057	
PPP	Glucose 6-phosphate	328.6	45.5	−86.1	0.038	↓
	6-Phospho-gluconic acid	269.9	607.7	125.1	0.727	
TCA	Malic acid	1126.1	514.0	−54.4	0.260	
	Succinic acid	250.5	168.8	−32.6	0.420	
	Fumaric acid	336.2	179.2	−46.7	0.260	
	cis-Aconitic acid	43.7	5.9	−86.4	0.050	↓
	Citric acid	6425.6	600.3	−90.7	0.050	↓
	alpha-Ketoglutaric acid	222.3	1818.6	718.3	0.483	
Urea Cycle	Citrulline	10.3	14.1	36.8	0.500	
	Arginine	14,526.1	4528.0	−68.8	0.420	
	Ornithine	2626.1	1223.7	−53.4	0.327	
AA	Proline	2642.0	2335.2	−11.6	0.841	
	Glutamine	31.0	25.8	−16.6	0.168	
	L-Glutamic acid	410.2	466.0	13.6	0.841	
Additional	Lactic acid	4613.3	17,831.6	286.5	0.038	↑
	Fructose	2.3	11.5	392.3	0.057	

**Table 3:** Targeted metabolic profiles of some metabolites of glycolysis, pentose phosphate pathway, TCA and urea cycles in MCF-7^crPOX^ treated with MET (MCF-7^crPOX+MET^) and in MCF-7^WT^ cultured in Gln free medium. AA—amino acids, PPP—pentose phosphate pathway, TCA—tricarboxylic acids cycle. ↑ —significant increase in the concentration of studied compound in MCF-7^crPOX+MET^ cells vs. MCF-7^WT^, ↓ —significant decrease in the concentration of studied compound in MCF-7^crPOX+MET^ cells vs. MCF-7^WT^.

Relevant Metabolic Pathways	Metabolites	Conc. Average (ppb)		% Change (MCF-7^crPOX+MET^ vs. MCF-7^WT^)	*P* Value (Mann–Whitney)	MCF-7^crPOX+MET^
		MCF-7^WT^	MCF-7^crPOX+MET^			
Glycolysis	Pyruvic acid	6.5	289.6	4378.6	0.022	↑
	Glucose	11.2	303.1	2618.3	0.022	↑
	Phospho-enolpyruvic acid	6449.0	0.0	−100.0	0.022	↓
PPP	Glucose 6-phosphate	328.6	18.8	−94.3	0.025	↓
	6-Phospho-gluconic acid	269.9	71.9	−73.4	0.104	
TCA	Malic acid	1126.1	133.0	−88.2	0.025	↓
	Succinic acid	250.5	134.1	−46.5	0.169	
	Fumaric acid	336.2	27.6	−91.8	0.025	↓
	cis-Aconitic acid	43.7	1.1	−97.6	0.022	↓
	Citric acid	6425.6	0.0	−100.0	0.022	↓
	alpha-Ketoglutaric acid	222.3	78.2	−64.8	0.132	
Urea Cycle	Citrulline	10.3	10.3	0.4	0.802	
	Arginine	14,526.1	2098.1	−85.6	0.118	
	Ornithine	2626.1	59.4	−97.7	0.025	↓
AA	Proline	2642.0	1244.8	−52.9	0.121	
	Glutamine	31.0	29.1	−6.1	0.578	
	Glutamic acid	410.2	78.6	−80.8	0.025	↓
Additional	Lactic acid	4613.3	21,161.8	358.7	0.022	↑
	Fructose	2.3	18.8	706.0	0.022	↑

**Table 4:** Targeted metabolic profiles of some metabolites of glycolysis, pentose phosphate pathway, TCA and urea cycles in PRODH/POX-knock out of MCF-7 cells (MCF-7^crPOX^) and wild type MCF-7 cells (MCF-7^WT^) cultured in medium containing Gln. AA—amino acids, PPP—pentose phosphate pathway, TCA—tricarboxylic acids cycle.

Relevant Metabolic Pathways	Metabolites	Conc. Average (ppb)		% Change (MCF-7^crPOX^ vs. MCF-7^WT^)	*P* Value (Mann–Whitney)	MCF-7^crPOX^
		MCF-7^WT^	MCF-7^crPOX^			
Glycolysis	Pyruvic acid	94.3	130.9	38.9	0.653	
	Glucose	10.3	59.5	474.9	0.075	
	Phospho-enolpyruvic acid	3605.0	78.2	−97.8	0.075	
PPP	Glucose 6-phosphate	184.4	131.9	−28.4	0.660	
	6-Phospho-gluconic acid	794.5	814.3	2.5	1.000	
TCA	Malic acid	1361.2	635.2	−53.3	0.172	
	Succinic acid	195.5	158.0	−19.2	0.660	
	Fumaric acid	402.0	184.4	−54.1	0.172	
	cis-Aconitic acid	79.6	48.2	−39.5	0.653	
	Citric acid	7462.9	4477.2	−40.0	0.536	
	alpha-Ketoglutaric acid	949.3	684.7	−27.9	0.660	
Urea Cycle	Citrulline	6.1	11.0	81.5	0.377	
	Arginine	10,138.1	6694.5	−34.0	0.660	
	Ornithine	3957.4	1510.7	−61.8	0.172	
AA	Proline	3288.6	3373.5	2.6	1.000	
	Glutamine	296.2	65.9	−77.7	0.075	
	Glutamic acid	369.4	250.6	−32.2	0.543	
Additional	Lactic acid	24,919.0	23,080.4	−7.4	1.000	
	Fructose	12.0	21.4	78.1	0.075	

**Table 5:** Targeted metabolic profiles of some metabolites of glycolysis, pentose phosphate pathway, TCA and urea cycles in MET treated wild type MCF-7 cells (MCF-7^WT+MET^) and in MCF-7^WT^ cells cultured in medium containing Gln. AA—amino acids, PPP—pentose phosphate pathway, TCA—tricarboxylic acids cycle. ↑ —significant increase in the concentration of studied compound in MCF-7^WT+MET^ cells vs. MCF-7^WT^, ↓ —significant decrease in the concentration of studied compound in MCF-7^WT+MET^ cells vs. MCF-7^WT^.

Relevant Metabolic Pathways	Metabolites	Conc. Average (ppb)		% Change (MCF-7^WT+MET vs.^ MCF-7^WT^)	*P* Value (Mann–Whitney)	MCF-7^WT+MET^
		MCF-7^WT^	MCF-7^WT+MET^			
Glycolysis	Pyruvic acid	94.3	201.7	114.0	0.132	
	Glucose	10.3	124.2	1100.6	0.050	↑
	Phospho-enolpyruvic acid	3605.0	1502.4	−58.3	0.176	
PPP	Glucose 6-phosphate	184.4	35.7	−80.6	0.165	
	6-Phospho-gluconic acid	794.5	79.2	−90.0	0.050	↓
TCA	Malic acid	1361.2	779.1	−42.8	0.248	
	Succinic acid	195.5	114.5	−41.4	0.165	
	Fumaric acid	402.0	239.1	−40.5	0.248	
	cis-Aconitic acid	79.6	5.7	−92.9	0.050	↓
	Citric acid	7462.9	613.7	−91.8	0.050	↓
	alpha-Ketoglutaric acid	949.3	1576.9	66.1	0.248	
Urea Cycle	Citrulline	6.1	6.3	2.9	0.952	
	Arginine	10,138.1	10963.0	8.1	0.578	
	Ornithine	3957.4	2019.8	−49.0	0.248	
AA	Proline	3288.6	4193.5	27.5	0.165	
	Glutamine	296.2	1666.2	462.4	0.050	↑
	Glutamic acid	369.4	941.7	154.9	0.050	↑
Additional	Lactic acid	24,919.0	15,892.6	−36.2	0.248	
	Fructose	12.0	9.8	−18.3	0.086	

**Table 6:** Targeted metabolic profiles of some metabolites of glycolysis, pentose phosphate pathway, TCA and urea cycles in MET treated wild type MCF-7 cells (MCF-7^crPOX+ MET^) and in MCF-7^WT^ cells cultured in medium containing Gln. AA—amino acids, PPP—pentose phosphate pathway, TCA—tricarboxylic acids cycle. ↑ —significant increase in the concentration of studied compound in MCF-7^crPOX+MET^ cells vs. MCF-7^WT^, ↓ —significant decrease in the concentration of studied compound in MCF-7^crPOX+MET^ cells vs. MCF-7^WT^.

Relevant Metabolic Pathways	Metabolites	Conc. Average (ppb)		% Change (MCF-7^crPOX+MET^ vs. MCF-7^WT^)	*P* Value (Mann–Whitney)	MCF-7^crPOX+MET^
		MCF-7^WT^	MCF-7^crPOX+MET^			
Glycolysis	Pyruvic acid	94.3	131.6	39.6	0.586	
	Glucose	10.3	204.6	1878.0	0.025	↑
	Phospho-enolpyruvic acid	3605.0	0.0	−100.0	0.025	↓
PPP	Glucose 6-phosphate	184.4	0.0	−100.0	0.025	↓
	6-Phospho-gluconic acid	794.5	0.0	−100.0	0.025	↓
TCA	Malic acid	1361.2	37.0	−97.3	0.025	↓
	Succinic acid	195.5	91.9	−53.0	0.086	
	Fumaric acid	402.0	6.1	−98.5	0.025	↓
	cis-Aconitic acid	79.6	1.4	−98.3	0.025	↓
	Citric acid	7462.9	0.0	−100.0	0.025	↓
	alpha-Ketoglutaric acid	949.3	40.9	−95.7	0.025	↓
Urea Cycle	Citrulline	6.1	1.9	−69.5	0.226	
	Arginine	10,138.1	539.3	−94.7	0.025	↓
	Ornithine	3957.4	0.0	−100.0	0.025	↓
AA	Proline	3288.6	3664.0	11.4	0.905	
	Glutamine	296.2	210.6	−28.9	0.461	
	Glutamic acid	369.4	68.4	−81.5	0.025	↓
Additional	Lactic acid	24,919.0	17,098.2	−31.4	0.226	
	Fructose	12.0	15.8	31.6	0.086	

## Data Availability

The datasets used and/or analyzed during the current study are available from the corresponding author on reasonable request.

## Supplementary Materials

The following are available online at https://www.mdpi.com/article/10.3390/biom11121888/s1, Figure S1: The PRODH/POX expression in MCF-7WT cells and MCF-7crPOX cells by Western Blot using Anti-PRODH/POX antibody (Santa Cruz).; Figure S2: The percentage of cells in G0/G1, S and G2/M phases of the cell cycle of MCF-7 and MCF-7crPOX cells treated with metformin with or without glutamine (Gln).; Figure S3: The representatives’ blots of AMPK expressions in MCF-7 cells and MCF-7crPOX cells treated with metformin (MET) cultured in DMEM in the presence and absence of glutamine.; Figure S4: The representatives’ blots of PRODH/POX expressions in MCF-7 cells and MCF-7crPOX cells treated with metformin (MET) cultured in DMEM in the presence and absence of glutamine.; Figure S5: The representatives’ blots of PARP and cleaved-PARP expressions in MCF-7 cells and MCF-7crPOX cells treated with metformin (MET) cultured in DMEM in the presence and absence of glutamine.; Figure S6: The representatives’ blots of Caspase-7 expressions in MCF-7 cells and MCF-7crPOX cells treated with metformin (MET) cultured in DMEM in the presence and absence of glutamine.; Figure S7: Representatives of chromatograms viewed by MassHunter Qualitative analysis navigator post-run LC-QqQ.; Figure S8: The results of lactic acid in Masshunter QQQ Quantitative analysis version 8.0 in reference samples (standard), testing sample and blank.; Table S1: Testing samples for MS-based approaches.; Table S2: The summary of testing metabolites.; Table S3: Optimized transition of targeted metabolites.
