# Supplementary material for: Metformin Treatment or PRODH/POX-Knock out Similarly Induces Apoptosis by Reprograming of Amino Acid Metabolism, TCA, Urea Cycle and Pentose Phosphate Pathway in MCF-7 Breast Cancer Cells
Source: Biomolecules. 2021 Dec 15;11(12):1888. doi: 10.3390/biom11121888 (PMC8699520; doi:10.3390/biom11121888)
Supplement: Supplementary file 1 [file biomolecules-11-01888-s001.zip › biomolecules-1489267-supplementary.pdf]

Supplementary Material

Metformin treatment or PRODH/POX-knock out similarly induces apoptosis by reprograming of amino acid metabolism, TCA, Urea cycle and pentose phosphate pathway in MCF-7 breast cancer cells.

Thi Yen Ly Huynh[1], Ilona Oscilowska[2], Jorge Sáiz[3], Magdalena Niziol[2], Weronika Baszanowska[1], Coral Barbas[3], and Jerzy Palka[1,*]

1 Department of Medicinal Chemistry, Faculty of Pharmacy, Medical University of Bialystok, 15-089, Bialystok, Poland; ly.huynhthiyen@umb.edu.pl (TYLH); w.baszanowska22@wp.pl (WB); pal@umb.edu.pl (JP).
2 Department of Pharmaceutical and Biopharmaceutical Analysis, Faculty of Pharmacy, Medical University of Bialystok, 15-089, Bialystok, Poland; ilona.zareba@gmail.com (IO), Magdalena.niziol@umb.edu.pl (M.N.)
3 Centre for Metabolomics and Bioanalysis (CEMBIO), University CEU San Pablo, 28003 Madrid, Spain; jorge.saizgalindo@ceu.es (JS); cbarbas@ceu.es (CB).
* Correspondence: Department of Medicinal Chemistry, Faculty of Pharmacy, Medical University of Bialystok, 15-089, Bialystok, Poland. Phone number: +48 85748 5706. E-mail: pal@umb.edu.pl (JP).

Supplementary Material

1. Calculation of percentage of change (%)

Due to zero value of several metabolites, the percentage of change (%) were calculated in different ways to avoid the non-valid % change.

- In Group B vs group G, (%) change was calculated by:

Change (%) = [(average of metabolite concentration in MCF-7^{WT} cells group – average of metabolite concentration in MCF-7^{crPOX} cells group)/[average of metabolite concentration in MCF-7^{crPOX} cells group] × 100

- In group D vs group I and group E vs group J, (%) change was calculated by:

Change (%) = [(average of metabolite concentration in MCF-7^{crPOX} cells group – average of metabolite concentration in MCF-7^{WT} cells group)/[average of metabolite concentration in MCF-7^{WT} cells] × 100

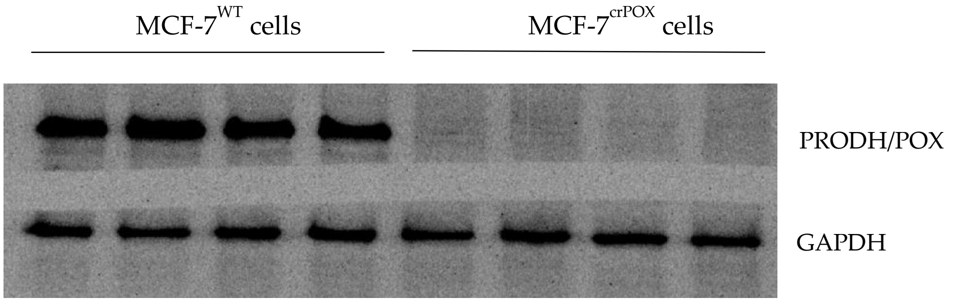

Figure S1. The PRODH/POX expression in MCF-7^{WT} cells and MCF-7^{crPOX} cells by Western Blot using Anti-PRODH/POX antibody (Santa Cruz).

2. Cell cycle phases

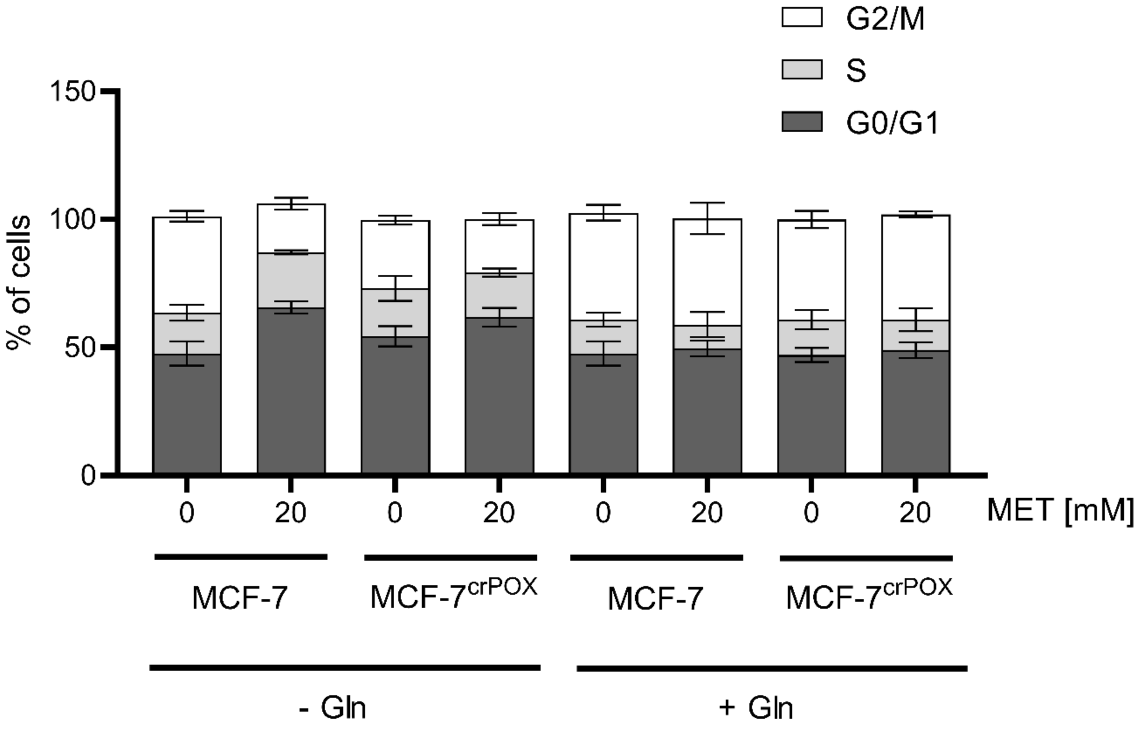

Figure S2. The percentage of cells in G0/G1, S and G2/M phases of the cell cycle of MCF-7 and MCF-7[crPOX] cells treated with metformin with or without glutamine (Gln).

3. **Expression of AMPPK**

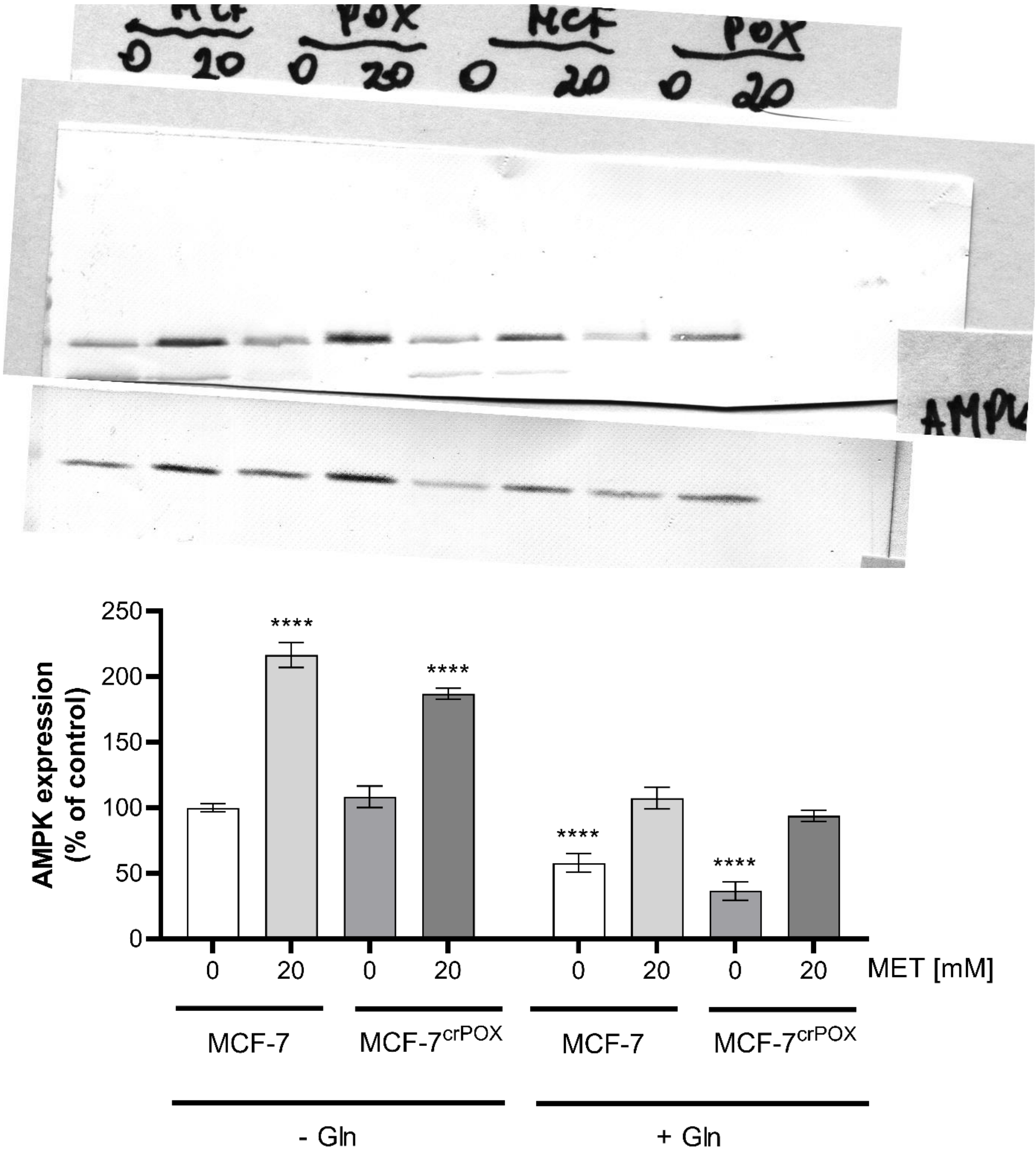

Figure S3. The representatives' blots of AMPK expressions in MCF-7 cells and MCF-7^{crPOX} cells treated with metformin (MET) cultured in DMEM in the presence and absence of glutamine. GAPDH expression was used as a loading control. The WB bands intensity of representative gels was quantified by densitometry and normalized to GAPDH. The densitometry values represent the mean (% of control) ± SD of three experiments, *P <0.001.

4. **Expression of PRODH/POX**

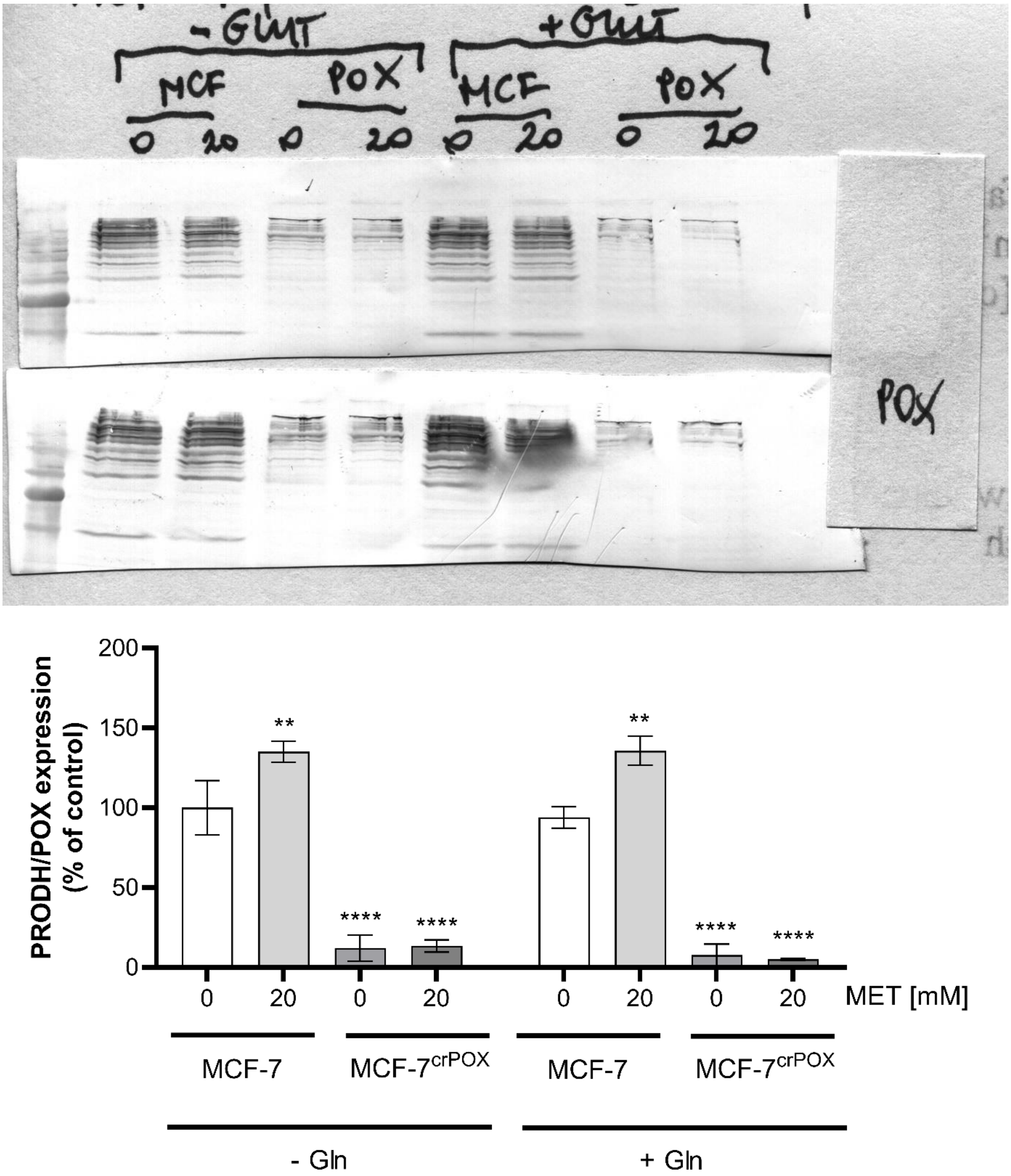

Figure S4. The representatives' blots of PRODH/POX expressions in MCF-7 cells and MCF-7^{crPOX} cells treated with metformin (MET) cultured in DMEM in the presence and absence of glutamine. GAPDH expression was used as a loading control. The WB bands intensity of representative gels was quantified by densitometry and normalized to GAPDH. The densitometry values represent the mean (% of control) ± SD of three experiments, *P <0.001.

5. **Expression of PARP and cleaved-PARP**

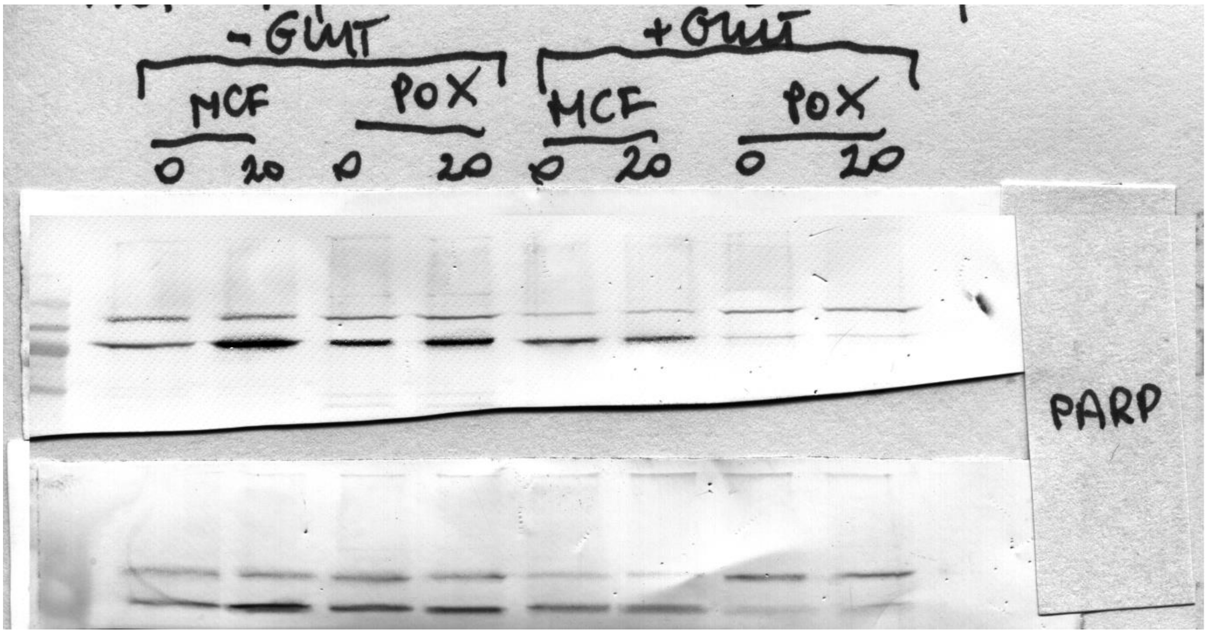

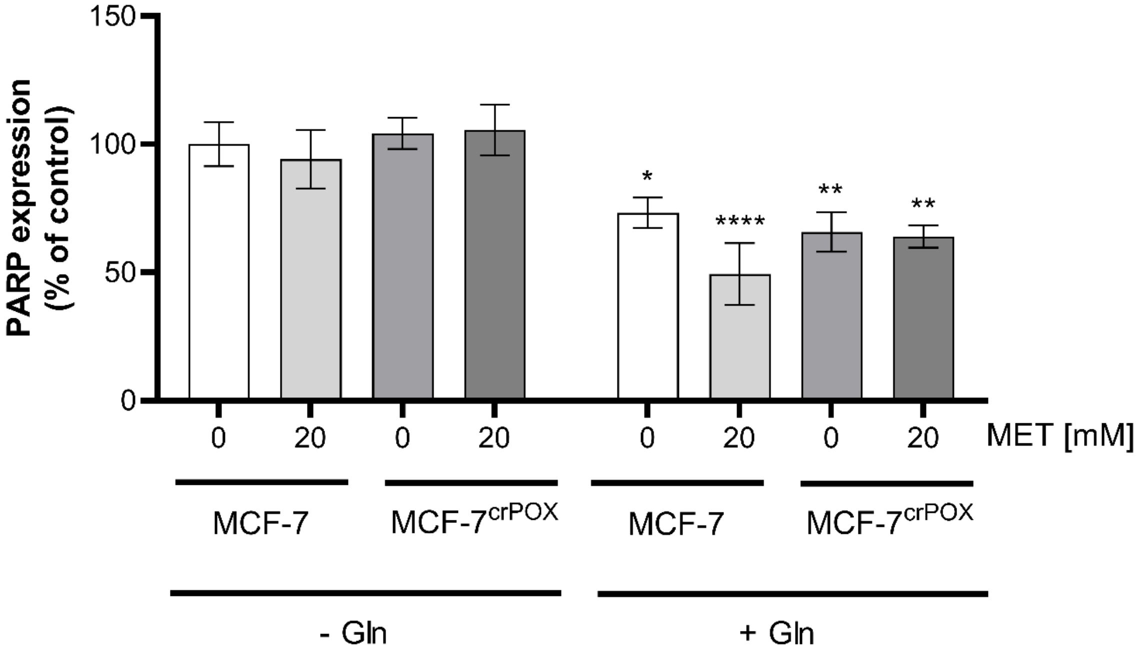

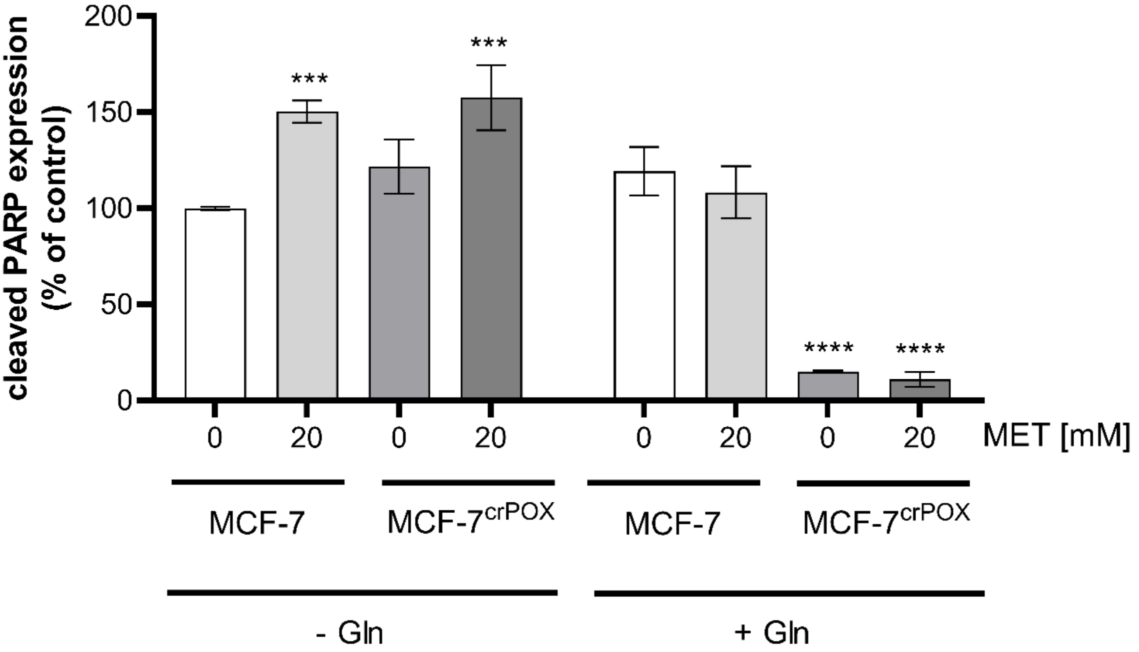

Figure S5. The representatives' blots of PARP and cleaved-PARP expressions in MCF-7 cells and MCF-7crPOX cells treated with metformin (MET) cultured in DMEM in the presence and absence of glutamine. GAPDH expression was used as a loading control. The WB bands intensity of representative gels was quantified by densitometry and normalized to GAPDH. The densitometry values represent the mean (% of control) ± SD of three experiments, *P <0.001.

6. Expression of Caspase-7

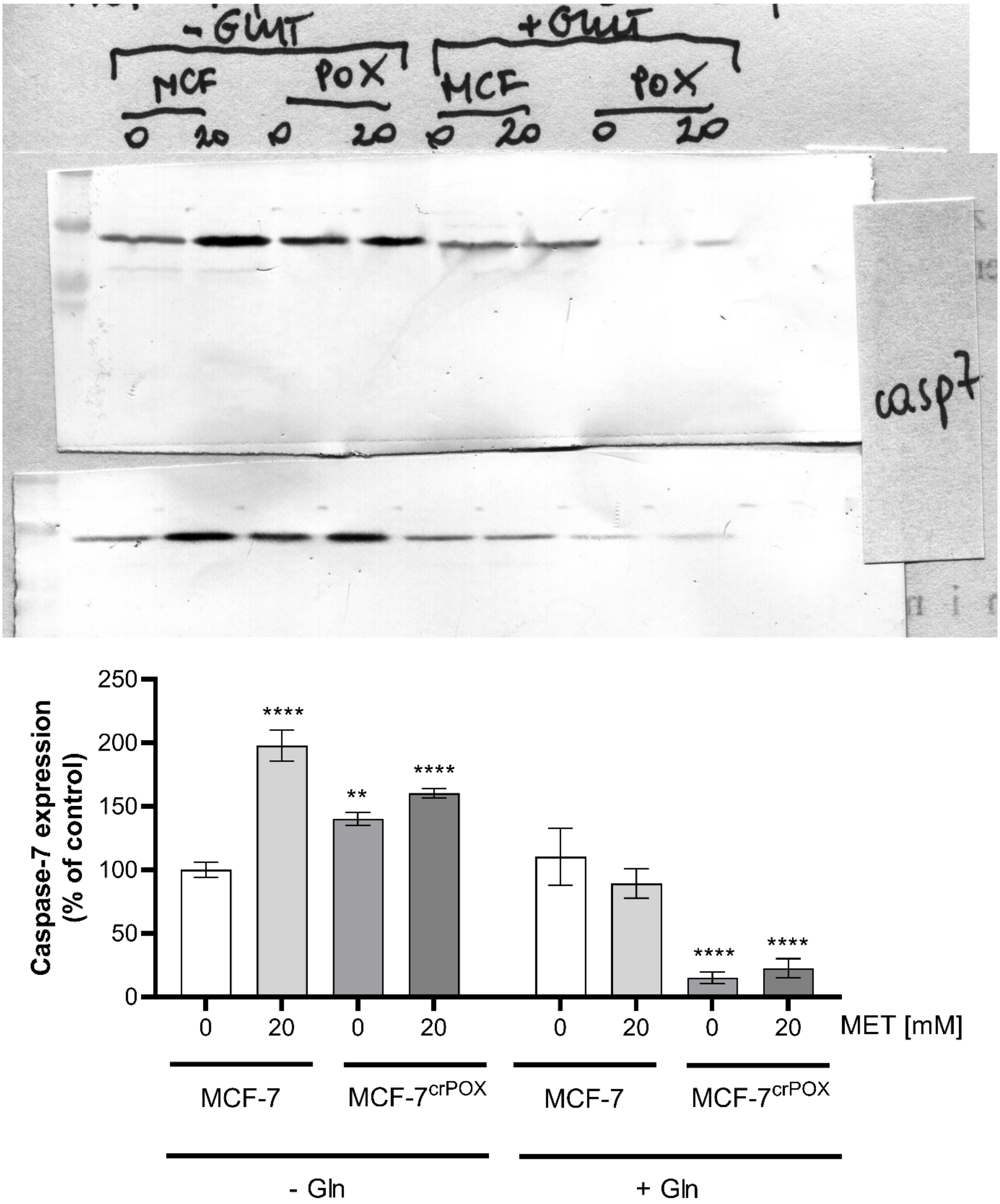

Figure S6. The representatives' blots of Caspase-7 expressions in MCF-7 cells and MCF-7[crPOX] cells treated with metformin (MET) cultured in DMEM in the presence and absence of glutamine. GAPDH expression was used as a loading control. The WB bands intensity of representative gels was quantified by densitometry and normalized to GAPDH. The densitometry values represent the mean (% of control) ± SD of three experiments, *P <0.001.

Table S1. Testing samples for MS-based approaches

No. Samples	Descriptions	Groups
1-5	MCF-7^{WT} cells in DMEM + glutamine, glucose 4g.l^{-1}, treated with Metformin 20 mM	A
6-10	MCF-7^{WT} cells in DMEM + glutamine, glucose 4g.l^{-1}, untreated	B
11-15	MCF-7^{WT} cells in DMEM - glutamine, glucose 4g.l^{-1}, treated with Metformin 20 mM	C
16-20	MCF-7^{WT} cells in DMEM - glutamine, glucose 4g.l^{-1}, untreated	D
21-25	MCF-7^{WT} cells in DMEM - glutamine, low glucose 1g.l^{-1}, untreated	E
26-30	MCF-7^{crPOX} cells in DMEM + glutamine, glucose 4g.l^{-1}, treated with Metformin 20 mM	F
31-35	MCF-7^{crPOX} cells in DMEM + glutamine, glucose 4g.l^{-1}, untreated	G
36-40	MCF-7^{crPOX} cells in DMEM - glutamine, glucose 4g.l^{-1}, treated with Metformin 20 mM	H
41-45	MCF-7^{crPOX} cells in DMEM - glutamine, glucose 4g.l^{-1}, untreated	I
46-50	MCF-7^{crPOX} cells in DMEM - glutamine, low glucose 1g.l^{-1}, untreated	J

MCF-7^{WT} cells: Wild type MCF-7 cells
MCF-7^{crPOX} cells: PROPDH/POX knockout MCF-7 cells

Table S2. The summary of testing metabolites

Relevant metabolic pathways	Metabolites
Glycolysis	Glucose
	Phosphoenol-pyruvic acid
	Pyruvic acid
Pentose phosphate pathway	Glucose 6-phosphate
	6-phospho-gluconate
Krebs cycle	Fumaric acid
	Alpha-ketoglutaric acid
	Citric acid
	Succinic acid
	Malic acid
	Cis-aconitic acid
Urea cycle	Ornithine
	Arginine
	Citrulline
Amino acids	Proline
	Glutamine
	Glutamic acid
Additional metabolites	Lactic acid
	Fructose

Table S3. Optimized transition of targeted metabolites

No.	Metabolites	Transition	No.	Metabolites	Transition
1	Glucose	259 → 79	11	Phosphoenolpyruvate	167 → 79
2	Fumaric acid	115.1 → 71	12	Pyruvic acid	87 → 43.2
3	Lactic acid	89 → 45.3	13	Succinic acid	117 → 73.1
4	Arginine	173.1 → 131	14	6- phosphogluconic acid	275.1 → 78.9
5	Citrulline	174 → 131	15	Alpha ketoglutaric acid	145 → 101
6	Glutamic acid	146 → 128	16	Cis-aconitic acid	173 → 129
7	Glutamine	145.1 → 127	17	Citric acid	191 → 111
8	Malic acid	133 → 115	18	Fructose	179.1 → 59
9	Ornithine	133 → 133	19	Glucose	179.1 → 59
10	Proline	114 → 68.1			

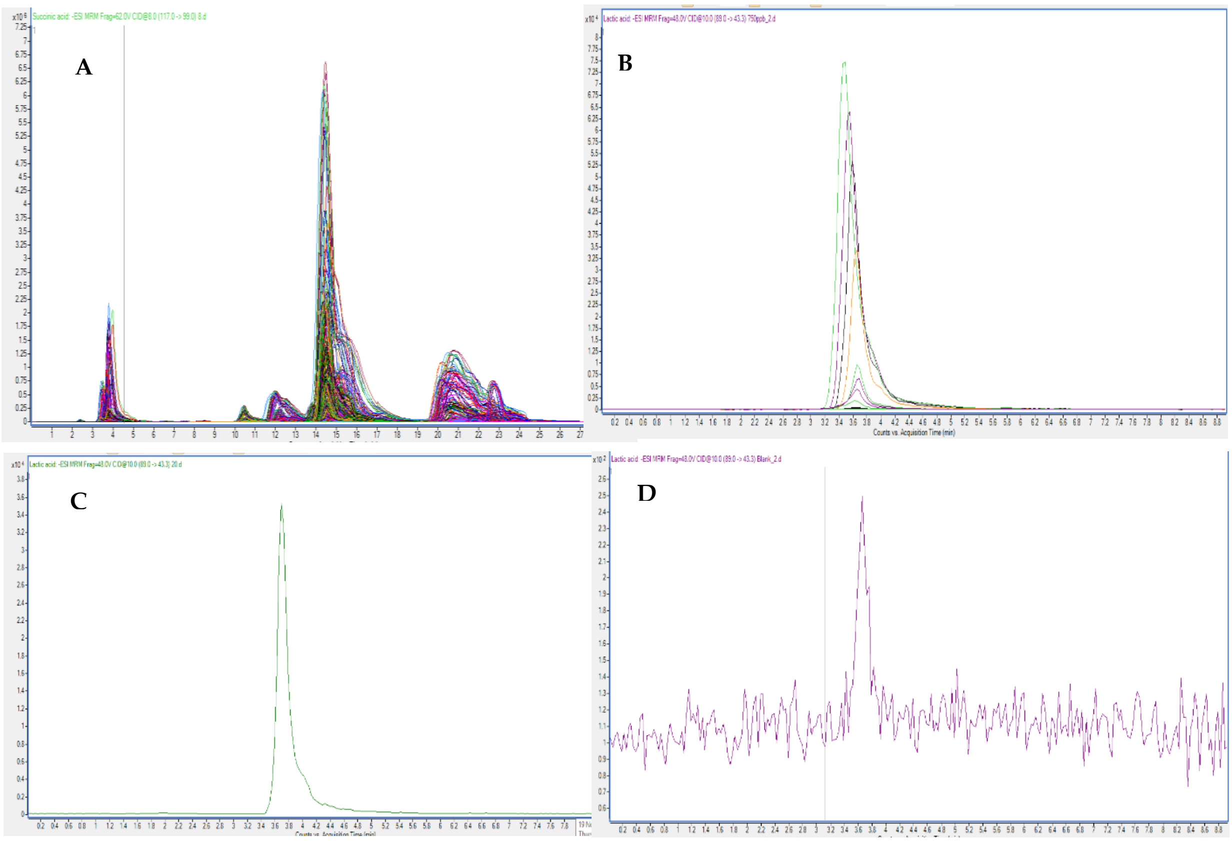

Figure S7. Representatives of chromatograms viewed by MassHunter Qualitative analysis navigator post-run LC-QqQ. A. All extracted profiles. **B.** The standard curve of Lactic acid. **C.** A testing sample with extracted Lactic acid peak. **D.** Blank.

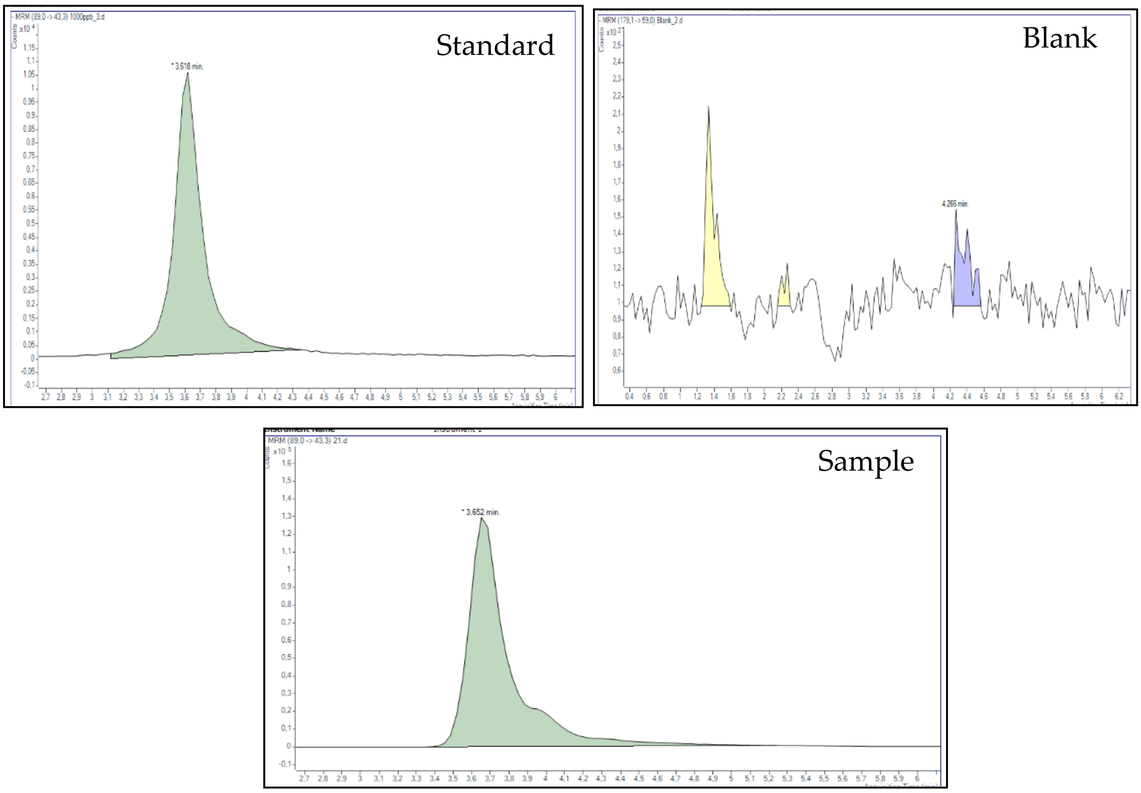

Figure S8. The results of lactic acid in Masshunter QQQ Quantitative analysis version 8.0 in reference samples (standard), testing sample and blank.
